# *Data Exploration Toolkit* for serial diffraction experiments

**DOI:** 10.1107/S1399004714025875

**Published:** 2015-01-23

**Authors:** Oliver B. Zeldin, Aaron S. Brewster, Johan Hattne, Monarin Uervirojnangkoorn, Artem Y. Lyubimov, Qiangjun Zhou, Minglei Zhao, William I. Weis, Nicholas K. Sauter, Axel T. Brunger

**Affiliations:** a Department of Molecular and Cellular Physiology, Stanford University, USA; b Howard Hughes Medical Institute, USA; c Physical Biosciences Division, Lawrence Berkeley National Laboratory, Berkeley, CA 94720, USA; d Janelia Research Campus, 19700 Helix Drive, Ashburn, VA 20147, USA; e Department of Structural Biology, Stanford University, USA; f Department of Photon Science, SLAC National Laboratory, Menlo Park, California, USA

**Keywords:** *Data Exploration Toolkit*, ultrafast diffraction, X-ray free-electron lasers

## Abstract

Ultrafast diffraction at X-ray free-electron lasers (XFELs) has the potential to yield new insights into important biological systems that produce radiation-sensitive crystals. An unavoidable feature of the ‘diffraction before destruction’ nature of these experiments is that images are obtained from many distinct crystals and/or different regions of the same crystal. Combined with other sources of XFEL shot-to-shot variation, this introduces significant heterogeneity into the diffraction data, complicating processing and interpretation. To enable researchers to get the most from their collected data, a toolkit is presented that provides insights into the quality of, and the variation present in, serial crystallography data sets. These tools operate on the unmerged, partial intensity integration results from many individual crystals, and can be used on two levels: firstly to guide the experimental strategy during data collection, and secondly to help users make informed choices during data processing.

## Abstract 2

This paper describes a set of tools allowing experimentalists insight into the variation present within large serial data sets.

## 1. Introduction   

Third-generation synchrotron light sources have been tremendously successful in tackling challenging problems in structural biology. Even with modern synchrotrons that deliver ∼10^12^ photons s^−1^, data collection still takes seconds to minutes per sample. Radiation damage during these exposures is a leading cause of failure in X-ray structure determination (Zeldin *et al.*, 2013 ▶). X-ray free-electron lasers (XFELs) have enabled a ‘diffraction before destruction’ approach that can circumvent this problem and provide effectively damage-free diffraction data sets (Chapman *et al.*, 2011 ▶; Kern *et al.*, 2013 ▶; Liu *et al.*, 2013 ▶; Hirata *et al.*, 2014 ▶). In addition, new sample-delivery and data-acquisition hardware have made it possible to perform ‘serial’ experiments with hundreds to thousands of crystals on both XFEL and modern synchrotron beamlines (Gati *et al.*, 2014 ▶). Understanding the causes of systematic variation in these increasingly used sources of data has general importance for biological crystallography.

One of the most significant challenges in analysing data from XFEL crystal diffraction experiments is data processing. XFEL data-processing efforts have so far focused on the essential task of processing individual images, and there has been considerable progress in this field (White *et al.*, 2012 ▶; Hattne *et al.*, 2014 ▶). The integrated intensities from all of these individual images are then merged in a single step, but so far no consideration is taken of the variation between the crystals, or between different regions of a single crystal, that constitute a complete diffraction data set. Sources of variation could include biologically significant alternate conformations of side chains and backbone, different configurations/conformations of multi-component macromolecular complexes, as well as the challenging issues of unit-cell variation, mosaicity and sample-to-sample variation in diffraction quality. We have developed a set of tools, the *Data Exploration Toolkit*, that provide experimentalists with immediate insight into their raw unmerged data in a form that is directly meaningful to users with synchrotron crystallo­graphy experience.

## 2. Methods   

### 2.1. Unit-cell variation   

Unit-cell variation is analyzed using a single-linkage hierarchical clustering procedure, with a user-defined threshold to select where branches of the tree are cut. In hierarchical clustering, the individual diffraction patterns are shown as leaves of the tree along the horizontal axis, and the effective distance between patterns, or clusters thereof, is shown along the vertical axis. The point at which two branches merge shows the differences between the members of each branch. The default distance metric is the Andrews–Bernstein NCDist metric (Andrews & Bernstein, 2014 ▶; McGill *et al.*, 2014 ▶), which uses manifold embedding to take symmetry into account when finding the shortest path within the Niggli cone between two sets of unit-cell parameters. Euclidian distance can optionally be used, resulting in less robust but faster results. Clustering was performed using the SciPy Python package (Oliphant, 2007 ▶).

### 2.2. Crystal orientations   

The direction of each real-space axis is projected onto a sphere, which is visualized as a two-dimensional map using the *Matplotlib BaseMap* package (http://matplotlib.org/basemap/). The averaged density of real space axes is also shown as a color map in order to aid visualization of the orientational distribution.

### 2.3. Intensity statistics   

Intensity statistics are calculated directly on the raw partially recorded integrated intensities. The scale (intercept) and gradient (−2*B*) are calculated by performing a linear regression on the log(*I*_partial_) *versus* [sin(θ)/λ]^2^ plots. The value of *B* obtained in this way is referred to as a ‘pseudo-Wilson’ statistic, since the intensities are partial and unnormalized.

### 2.4. Example data   

Example data were collected using a goniometer setup on the XPP beamline of the LCLS (Cohen *et al.*, 2014 ▶) from crystals of a macromolecular complex being studied in the Brunger group (manuscript in preparation), mounted in loops and cryocooled. Beam-energy metadata was added from the XTC stream using *cctbx.xfel* and the resulting image files were integrated using the *cxi.index* program from *cctbx.xfel* (Hattne *et al.*, 2014 ▶).

## 3. Overview of the toolkit   

The intended use of the *Data Exploration Toolkit* is to provide experimentalists with feedback on the heterogeneity and quality of their unmerged diffraction data. Performing these analyses before merging is key. For example, a poor correlation coefficient or *R* factor in the merging statistics does not provide any guidance regarding the source of these low scores: were all of the crystals equally bad, or were the crystals made up of individual populations whose diffraction patterns may merge well separately but when taken together have poor internal consistency?

The toolkit is implemented as a set of four command-line programs accessible to users of *cctbx.xfel* (see Table 1 ▶). The unit-cell clustering tool, *cluster.unit_cell*, provides a fast way to perform and visualize hierarchical clustering on the unit-cell dimensions, using either the symmetry-aware Andrews–Bernstein distance (Andrews & Bernstein, 2014 ▶; McGill *et al.*, 2014 ▶) or a simple Euclidean distance metric. The orientational bias tool, *cluster.visualize_orientations*, allows a direct, laboratory-frame visualization of the orientation of the crystals; this makes it simple to visualize any systematic alignments of crystals. Because this tool allows experimentalists to identify bias quickly (typically within minutes), it is possible to decide to skip a sample if there is significant bias, allowing more efficient use of precious XFEL beamtime. The overall intensity tool, *cluster.intensity_statistics*, displays a pseudo-Wilson plot of the partial intensities of all the images against resolution, and the per-frame intensity tool, *cluster.individual_frame_intensity*, shows the distribution of partial intensities on a frame-by-frame basis. The intensity tools can help to identify anomalies in the diffraction intensities for a subset of images before merging. Outliers could be caused by either mis-indexing or by poor diffraction; a simple test to distinguish between these causes would be to repeat the intensity analysis on a highly isomorphous subset of diffraction images that was identified using the unit-cell clustering tool. Finally, the outputs of these four tools can be shown within a single figure for a quick overview of the current state of the diffraction data using the command *cluster.42*, providing unit-cell distributions, crystal orientations and the distribution of partial intensity reflections *versus* resolution. Each program acts on a folder of *cctbx.xfel* image files, and additional parameters can be specified *via* the command line.

**Figure 1:**
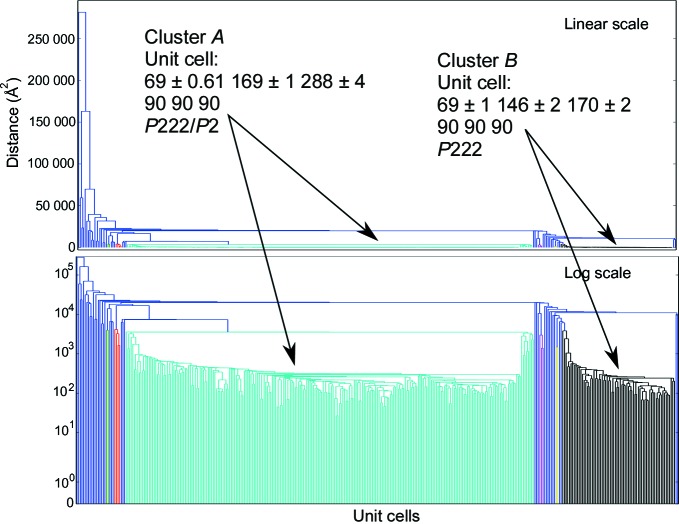
Hierarchical clustering of the test data, with both a linear (top) and a log (bottom) *y* axis. Each branch of the tree below the threshold (5000 Å^2^) is defined as a cluster and colored individually. Single-element clusters are labeled in blue. The two crystal forms are shown in green and black, representing the long-cell and short-cell forms, respectively. The median of each cluster can then be used as a target in order to obtain significantly higher indexing rates.

**Figure 2:**
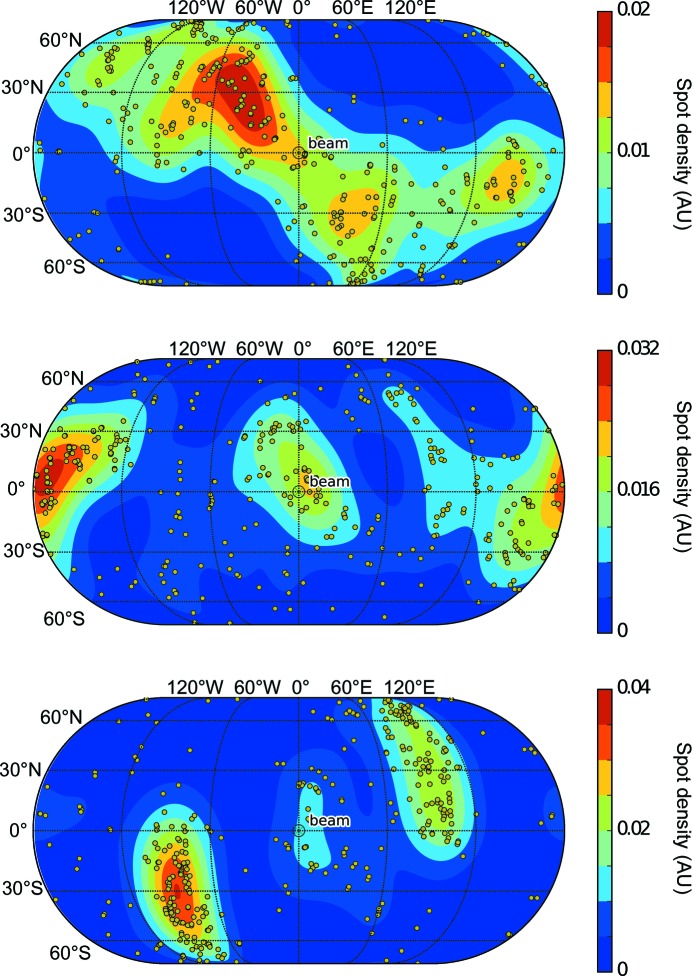
Orientational distribution of real-space axes (*a* axis, top; *b*, middle; *c*, bottom) from the test sample, showing a significant amount of bias, probably owing to the preferred orientations of a crystal within the loop. The unit vector describing the direction orientation of the crystal axis is projected onto a unit sphere, and its orientation relative to the beam is shown in terms of latitude/longitude for ease of interpretation. Thus (0, 0) is along the beam, North/South represent up/down and East/West represent right/left. This tool is complementary to the *PHENIX* reflection viewer (*phenix.data_viewer*; Adams *et al.*, 2010 ▶), which visualizes any missing wedges in reciprocal space, since it provides a direct reference back to the laboratory frame, allowing experimental adjustments to be made where possible. For each crystal, the laboratory-frame direction of the three real-space axes is shown as a yellow spot. The averaged density of real space axes is added to help in the interpretation of trends.

**Figure 3:**
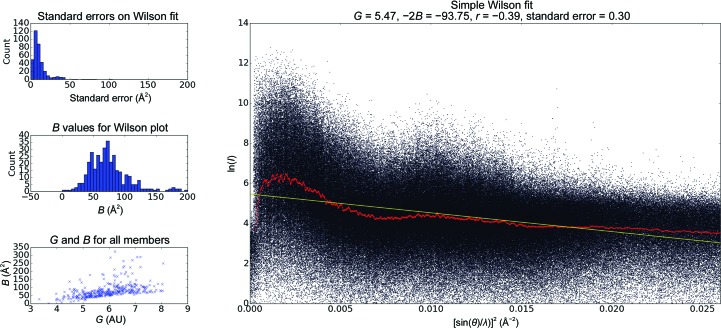
Intensity statistics for the partial unmerged data integrated without a target cell. The main plot shows a monotonically decreasing trend for intensities as a function of increasing scattering angle. The plots on the left, aggregated from the per-frame pseudo-Wilson plots (examples are shown in Supplementary Fig. S2), show a number of outliers, consistent with the presence of outliers in Fig. 1 ▶. A similar plot, but for when the long cell was used as a crystal target, exhibits fewer outliers and is shown as Supplementary Fig. S3. Extreme values of either *G* (scale factor; intercept in the main plot) or −2*B* (the slope in the right-hand plot) may be caused by mis-indexing, and a filter to remove these can be applied by using the clustering toolkit from within a Python script within the *cctbx.xfel* environment.

As an example, the *Data Exploration Toolkit* was used on a recently collected goniometer-based data set of 789 images, where several diffraction patterns could be obtained from most crystals using femtosecond XFEL pulses, as detailed in §2.4. After an initial integration run, 367 images were indexed. Applying the unit-cell clustering tool to these images revealed two significant groups of indexing solutions (Fig. 1 ▶) containing 249 and 69 members, respectively. These were associated with two different known crystal forms, referred to as the ‘long’ and the ‘short’ unit cells, respectively. Both unit cells had ortho­rhombic (*P*222) lattices, with median unit-cell edges of (69, 169, 288) Å for the long cell and (69, 146, 170) Å for the short unit cell (the full clustering log file is shown in the Supporting Information). The diffraction data were then re-integrated using the long cell as a target, leading to 443 frames being indexed. A second round of clustering on these new integration results revealed that 427 of those were in a single cluster (Supplementary Fig. S1*a*), and these were then used for the merging steps. Using the short unit cell as a target led to 97 images being indexed, with 93 of these in a tight cluster (Supplementary Fig. S1*b*). An increase in effectively indexed images of 50–100% is typical when using an optimal target cell determined in this way. Upon inspection of the crystal orientations of the entire data set (Fig. 2 ▶), we observed significant bias consistent with the observations made while analyzing the incoming diffraction patterns during the data collection, which indicated that large crystals on the goniometer setup tended to have a preferred orientation in the loop. Finally, examining the pseudo-Wilson plot (Fig. 3 ▶) shows that there are no serious anomalies in the diffraction data (for example increasing intensities at high resolution) that could be indicative of sub-optimal integration parameters. The distribution of per-frame temperature factors and standard errors (from the per-frame partial intensity analysis; examples shown in Supplementary Fig. S2) also do not show many outliers or multimodal distributions.

## 4. Conclusions   

Using the *Data Exploration Toolkit* to identify the multiple crystal forms present in a particular crystallographic experiment, and to guide the choice of target unit cells, increased the number of successfully indexed images. The other tools in the suite allowed us to understand the orientation bias in the experimental setup and to ‘sanity-check’ the integrated intensities prior to merging. Taken together, these tools provide a quick, easy and valuable addition to the quiver of methods available to experimentalists when working with challenging serial data sets about which little is known.

The tools presented in this work allow experimentalists to gain valuable insights into the heterogeneity of their serial data and to obtain rapid feedback on spot-finding and integration parameters during data collection. Our tools extend those of the *CrystFEL* suite *cell_explorer* tool that visualizes individual unit-cell parameter histograms (White *et al.*, 2012 ▶). When this information is considered along with more traditional merging statistics, a clearer picture of data quality is available than when using either tool independently. These methods are thus applicable on two levels. Firstly, during experiments they can be used to help identify pathologies such as orientation bias or misleading hit rates. Given the current extreme scarcity of XFEL beamtime, this approach will also be helpful in guiding decisions about when to move on to another sample or when to keep going. Secondly, during data processing these tools provide rapid and valuable feedback on the quality of the unmerged data. The functionality provided by the command-line tools is also accessible from an application programming interface, and is therefore highly extensible and customizable for use during data processing by advanced users. This interface allows one to apply filters or select individual clusters for further processing using short, concise Python scripts. We hope that these fast, simple-to-use data-exploration tools will enable the efficient measurement of higher quality serial crystallography diffraction data.

## Supplementary Material

Supporting Information.. DOI: 10.1107/S1399004714025875/rr5088sup1.pdf

## Figures and Tables

**Table 1:** The five command-line applications in the *Data Exploration Toolkit*

*cluster.unit_cell*	Uses hierarchical clustering to visualize and cluster the unit cells output from the integration step.
*cluster.visualize_orientations*	Visualizes the orientational distribution of the real-space crystal axes, revealing any bias that may be present. Laboratory-frame orientations of the *a*, *b* and *c* axes are projected onto a globe, with a color map added to make the distribution more clear.
*cluster.intensity_statistics*	Aggregates data of partial intensities over all images. Generates a scatter plot of slope and intercept for all images, and histograms of gradient and standard errors on the fits. Also creates a super-plot of all of the partial log intensities *versus* sin^2^()/^2^.
*cluster.individual_frame_intensity*	Generates a plot of log(*I*_partial_) *versus* sin^2^()/^2^ for each image. Also plots a rolling average and a linear fit to the data: a ‘pseudo-Wilson’ plot.
*cluster.42*	Convenience utility to provide an aggregate of the unit cell, orientations and intensity histograms in a single frame with a single command.
