# Supplementary material for: Data Exploration Toolkit for serial diffraction experiments
Source: Acta Crystallogr D Biol Crystallogr. 2015 Jan 23;71(Pt 2):352–6. doi: 10.1107/S1399004714025875 (PMC4321488; doi:10.1107/S1399004714025875)
Supplement: Supplementary file 1 [file d-71-00352-sup1.pdf]

Acta Cryst D

BIOLOGICAL CRYSTALLOGRAPHY

Volume 71 (2015)

Supporting information for article:

Data Exploration Toolkit for serial diffraction experiments

Oliver B. Zeldin, Aaron S. Brewster, Johan Hattne, Monarin Uervirojnangkoorn, Artem Y. Lyubimov, Qiangjun Zhou, Minglei Zhao, William I. Weis, Nicholas K. Sauter and Axel T. Brunger

Full unit cell output:

```
INFO cluster.ab_cluster: Using Andrews-Bernstein distance from Andrews & Bernstein J Appl Cryst 47:346 (2014)
44 clusters.
C_id        Num in cluster Med_a       Med_b       Med_c       Med_alpha    Med_beta     Med_gamma
cluster_39       2         167.9(0.7)  68.9 (0.0)  286.5(1.5)  90.00 (0.00) 90.93 (0.15) 90.00 (0.00)
2 in P2.
cluster_40       2         68.4 (0.1)  147.3(0.6)  168.7(1.7)  90.00 (0.00) 90.06 (1.23) 90.00 (0.00)
2 in P2.
cluster_41       3         69.9 (1.1)  168.5(1.9)  153.7(1.1)  90.00 (0.00) 94.41 (0.28) 90.00 (0.00)
3 in P2.
cluster_42       4         68.3 (1.0)  169.5(1.4)  284.9(2.7)  90.00 (0.00) 90.72 (0.88) 90.00 (0.00)
4 in P2.
cluster_43       69        68.8 (0.9)  146.2(3.0)  169.5(3.1)  90.00 (0.00) 90.00 (0.26) 90.00 (0.00)
67 in P222, 2 in P2.
cluster_44      249        68.6 (6.6)  169.2(25.3) 288.2(25.0) 90.00 (0.00) 90.00 (0.25) 90.00 (0.00)
237 in P222, 12 in P2.
Standard deviations are in brackets.
38 singletons:

 Point group   a           b           c           alpha        beta         gamma
C2            62.9        167.8       563.2       90.0         90.0         93.3
C222          65.9        168.2       556.5       90.0         90.0         90.0
C222          69.4        164.7       501.1       90.0         90.0         90.0
P2            70.8        169.2       291.5       90.0         94.0         90.0
P2            70.1        166.5       305.2       90.0         94.1         90.0
P1            67.0        166.5       268.3       92.0         94.2         92.5
P1            66.7        166.9       283.0       92.3         93.4         92.5
P1            79.9        173.1       289.4       87.4         85.7         82.5
C2            67.7        228.2       242.2       90.0         90.0         93.0
C2            67.6        237.5       248.4       90.0         90.0         92.8
P2            70.0        176.4       285.7       90.0         90.0         93.1
P2            65.2        169.8       289.5       90.0         90.0         93.4
P2            68.5        169.3       287.0       90.3         90.0         90.0
P2            68.7        170.1       289.3       90.3         90.0         90.0
P2            68.6        169.4       289.1       90.5         90.0         90.0
P222          68.4        169.4       255.6       90.0         90.0         90.0
P222          68.4        169.9       267.0       90.0         90.0         90.0
P2            59.8        168.8       268.2       90.0         90.0         91.6
P2            67.5        147.3       165.1       90.0         95.2         90.0
P2            66.4        144.8       168.9       90.0         90.0         91.7
P2            67.4        143.7       168.6       90.0         90.0         92.6
P2            55.2        140.6       149.2       108.7        90.0         90.0
P2            69.2        118.7       170.0       96.6         90.0         90.0
P2            69.2        85.7        167.7       96.6         90.0         90.0
P2            70.1        75.9        165.5       90.0         90.0         102.4
P1            70.1        152.3       171.0       86.9         86.7         87.1
P1            41.7        61.9        165.6       87.3         87.4         84.8
P2            67.7        145.6       169.6       90.5         90.0         90.0
P1            54.0        67.4        154.9       85.0         86.5         76.7
P1            71.2        173.8       182.5       114.5        100.3        94.9
P222          69.8        166.9       215.8       90.0         90.0         90.0
P2            68.7        166.9       200.2       90.0         94.3         90.0
P1            67.0        164.9       270.9       93.0         93.9         92.9
P1            66.2        133.0       167.6       94.8         93.4         97.9
P2            68.8        160.7       288.3       92.6         90.0         90.0
C2            68.9        167.7       217.4       99.6         90.0         90.0
C2            65.6        290.9       318.1       93.2         90.0         90.0
I222          68.5        159.4       772.2       90.0         90.0         90.0
```

Example output for the run of `cluster.unit_cell` on the test data, showing the two main clusters in bold. For each cluster, the cluster name, which is associated with a file containing the names of the integration files making up the cluster, number of images in the cluster, and median unit cell (with standard deviation) are specified. Clusters of size 1 (singletons) are shown, together with their unit cells and point groups below the main results.

Figures:

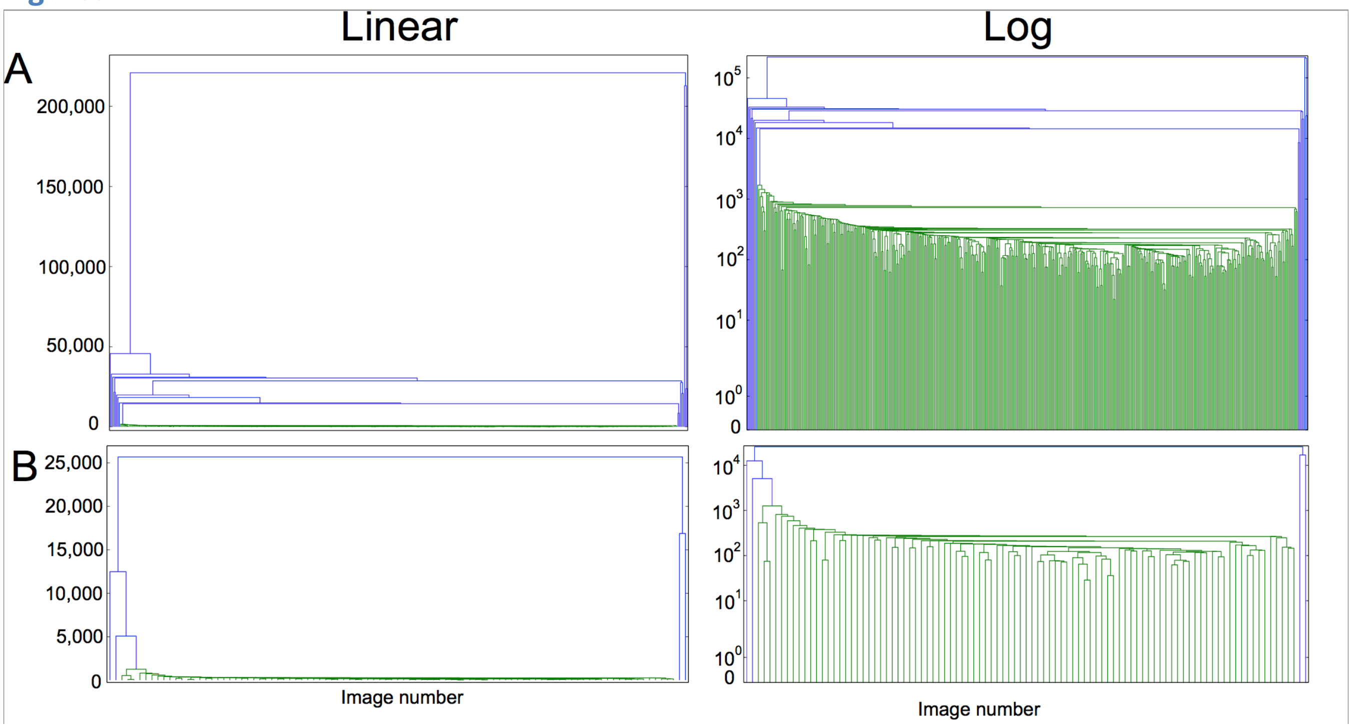

Supplementary figure 1. Unit cell clustering with targets. A) The 789 original test images were reprocessed with a new target (long cell, (60, 169, 288) Å) derived from the clustering results shown in Figure 2. The 443 resultant unit cells were clustered using `cluster.unit_cell` and the linear (left) and log (right) plots are shown, forming a single, tightly grouped cluster around the target used, and an increased indexing rate, as described in the main text. B) As A), using the short cell target ((69, 146, 170) Å). In Both cases, the *y* axis represents the distance between unit cells in Å^2.

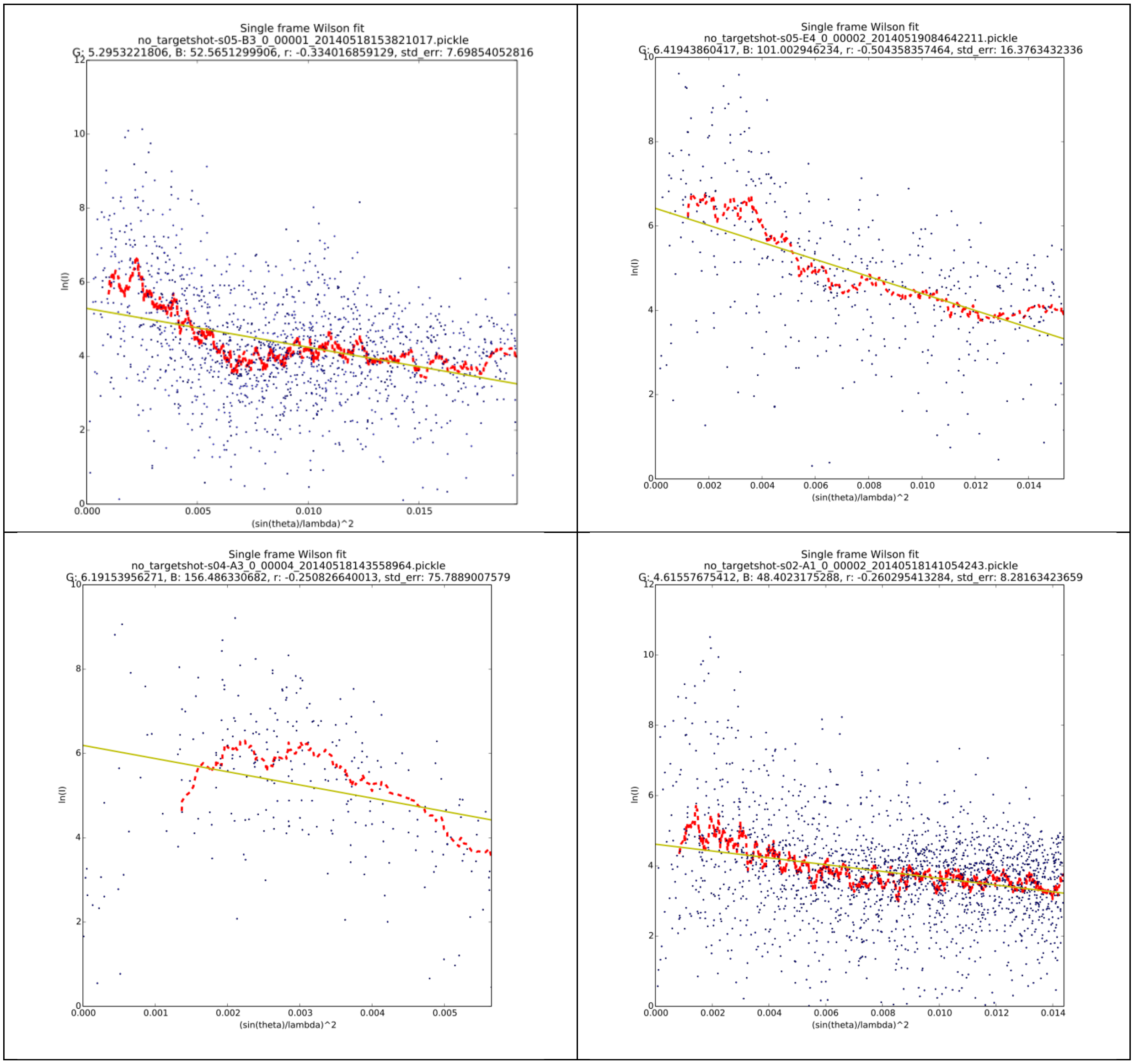

Supplementary figure 2 Example frames from `cluster.individual_frame_intensity`, showing the plot of log($I_{partial}$) vs. $\sin^2(\theta)/\lambda^2$ for all partial observations on the frame. A rolling average of the partial intensities is shown as a red line, and a linear fit to all the data in yellow. The intercept (G), slope (-2×B), *r* statistic and standard error on the fit are shown in the plot title.

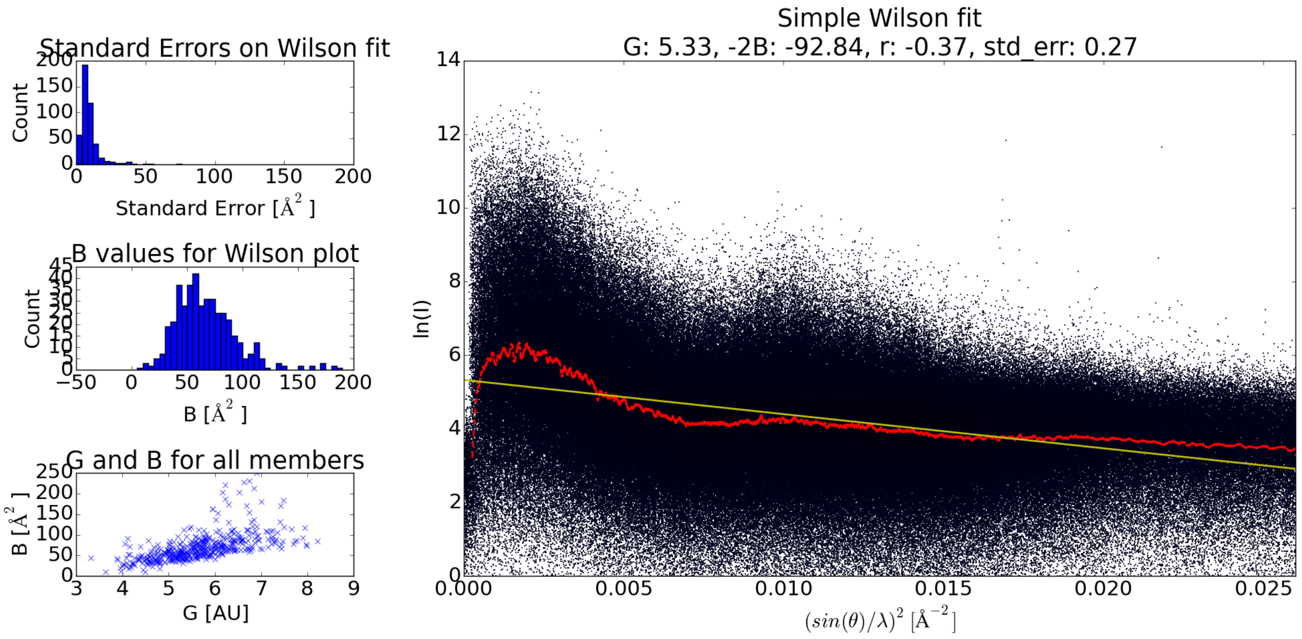

Supplementary figure 3. Full output example from `cluster.intensity_statistics` for the long cell crystal form. The main plot is denser than figure 3, due to more images being integrated with the long cell target, and so more reflections being plotted. In addition, there are fewer and less severe outliers in the aggregate data shown in the left three panels, reflecting the tighter cluster observed in the unit cell clustering shown in supplementary figure 1a.
